# Zinc nutritional status, mood states and quality of life in diarrhea-predominant irritable bowel syndrome: a case–control study

**DOI:** 10.1038/s41598-022-15080-2

**Published:** 2022-06-29

**Authors:** Mahsa Rezazadegan, Farnaz Shahdadian, Maryam Soheilipour, Mohammad Javad Tarrahi, Reza Amani

**Affiliations:** 1 Department of Clinical Nutrition, School of Nutrition and Food Science, Isfahan University of Medical Sciences, Isfahan, Iran; 2 Isfahan Gastroenterology and Hepatology Research Center (IGHRC), Isfahan University of Medical Sciences, Isfahan, Iran; 3 Department of Epidemiology and Biostatistics, School of Public Health, Isfahan University of Medical Sciences, Isfahan, Iran

**Keywords:** Psychology, Biomarkers, Gastroenterology

## Abstract

Zinc is an important trace element for structure, and regulation in the central nervous system, as well as the gut homeostasis. There are several mental disorders associated with zinc deficiency. The relationship between zinc nutritional status with mood states and quality of life (QoL) in diarrhea-predominant irritable bowel syndrome (IBS-D) has not been studied yet. This case–control study aimed to investigate the association between zinc nutritional status with mood states and QoL in IBS-D patients. Sixty-one newly diagnosed patients with IBS-D and 61 matched healthy controls were enrolled. Dietary zinc intakes and serum zinc levels were measured. Mood states and QoL were evaluated by validated questionnaires. Logistic regression was used to estimate the odds of IBS-D in relation to zinc deficiency. Decreased serum zinc levels were observed in the IBS-D group than in the controls (p = 0.001). There were higher scores of depression (p = 0.014), anxiety (p = 0.005), and stress (p = 0.001) among IBS-D patients. Moreover, overall QoL, physical and psychological health were lower in IBS-D patients compared to the controls (p < 0.001). “Food avoidance” had the lowest, while the “relationship” had the highest score among the patients (51.09 ± 26.80 and 78.14 ± 23.30, respectively). Dietary zinc intake was positively correlated with psychological health in the controls (r = 0.295, p = 0.022) and with body image in the patients (r = 0.266, p = 0.044). According to the logistic regression, zinc deficiency was not significantly associated with odds of IBS-D. Findings show that zinc deficiency may be associated with some parameters of IBS-D. Further clinical studies are needed to explore the causal relationship between zinc status and IBS pathogenesis.

## Introduction

Irritable bowel syndrome (IBS) is a functional gastrointestinal (GI) disorder with abdominal pain and alterations in stool frequency or form^1^. It can affect anyone, regardless of age, sex, or race^2^. Nevertheless, IBS is more common among people with psychological disorders and young adult women than among those in the general population^3^. Based on a previous meta-analysis, global estimation of the IBS prevalence is 11%^4^. Anxiety, depression, life events and stress, frequent healthcare use, and sleep disorders are the most common risk factors for IBS^5^. IBS is accompanied by depression or anxiety in 40–60% of cases, and these patients have a high frequency of somatic symptoms. Mood disorders, the severity of GI symptoms, and somatization are contributed to the reduced quality of life (QoL) in IBS patients^6^. Although IBS is not related to higher mortality rates, considering medical costs and reduced health-related QoL, it imposes significant burden on patients and society^7^. A healthy diet and appropriate nutrition not only contribute to physical health but also mental well-being and mood stability^8^.

Zinc is one of the most important nutrients which has significant importance for public health. It plays a critical role in biochemical pathways, such as response to oxidative stress, homeostasis, and immunological responses^9^. Almost 17.3% of people in the world suffer from zinc deficiency, which affects many organs^10^. Zinc is essential for neuronal impulses, and its deficiency impacts on mental wellbeing, brain growth, and behavior. The alteration of brain zinc levels has been implicated in a wide range of diseases such as Alzheimer's disease, Parkinson's disease, and mood disorders including depression^11,12^. A study showed subjects with anxiety had significantly higher plasma copper and lower zinc levels in comparison to their controls^13^. Moreover, a recent study found that serum zinc concentration is a useful marker of reduced health-related QoL in patients with chronic liver diseases^14^. Previous studies have indicated that there is a lower intake or serum zinc levels in patients with IBS but they did not examine its relation with mood and QoL^15–17^. Therefore, this case–control study purposed to evaluate the correlation between zinc nutritional status with mood states and QoL and the association of zinc deficiency with odds of IBS-D in diarrhea-predominant patients with irritable bowel syndrome (IBS-D).

## Subjects and methods

### Participants

The present case–control study was conducted on 61 IBS-D patients and 61 healthy matched controls, from April 2020 to January 2021, at Isfahan University of Medical Sciences Khorshid and Al-Zahra Hospital clinics. Based on Rome IV criteria, a gastroenterologist diagnosed new cases of IBS-D patients^18^. Study participants were men and women aged 20–55 with a body mass index (BMI) below 30 kg/m^2^. The exclusion criteria were: any evidence of abdominal surgery, celiac or other primary GI diseases and infection, pregnancy, lactation, malignancy, routine consumption of artificial sweeteners^19^, smoking and alcohol drinking habit, chronic anemia, concurrent chronic diseases, taking medications including glucocorticoids, non-steroidal anti-inflammatory drugs, antibiotics, antidiarrheal and antipsychotic drugs, having special diet or taking dietary supplements such as zinc during the last six months as well as being a professional athlete. Individuals without IBS (according to the gastroenterologist diagnosis) were selected among friends of the patients and were considered as the healthy controls. The frequency matching method was used to match the cases and controls based on their age and sex. The study was conducted according to the declaration of Helsinki and the STROBE checklist. All participants provided written informed consent and the study protocol was approved by the Medical Ethics Committee at the Isfahan University of Medical Sciences (reg. no. IR.MUI.RESEARCH.REC.1398.826).

### Demographic and anthropometric assessment

A general questionnaire was used to assess the educational level, employment, age, and drug history. Height was measured at a standing position without shoes using a fixed non-stretchable tape at 0.1 cm precision. Omron BF511 set was used to measure weight in minimal clothing. Waist circumference at the midpoint between the iliac crest and the last rib was assessed. The hip circumference was measured around the widest part of the buttocks. Body mass index was computed as dividing weight (kg) by height (m^2^).

### Dietary assessment

Dietary intake of all participants were gathered using a 3-day dietary record (two working days and one weekend day)^20^. We converted the portion sizes of foods consumed into grams. Customized Nutritionist IV software (First Databank Inc., Hearst Corp., San Bruno, CA, USA) was used for dietary analysis based on Iranian foods.

### Biochemical assessment

After 10–12 h of fasting, blood samples were collected from the cubital vein in all participants. Serums were separated, then were stored at − 80 °C until further assays. Serum zinc levels were measured using the colorimetric method (Zinc assay kit, Kiazist, Hamedan, Iran). The accuracy of this kit is close to the results obtained by atomic absorption spectrophotometry method^21^. We considered serum zinc levels less than 70 µg/dL as zinc deficiency^22^.

### Mood states

Participants were evaluated for depression, anxiety and stress scores using a 21-item questionnaire (DASS-21). Validation of this questionnaire was investigated previously among the Iranian population^23^. There were four response options, ranging from 0 (did not apply to me at all) to 3 (applied to me most of the time). Afterward, based on the scores in each question, each parameter of depression, anxiety, and stress was calculated.

### Quality of life

We used two questionnaires to assess the QoL. A self-report 34-item IBSQoL questionnaire was provided to IBS patients^24^. The Persian version of this instrument is reliable and validated having high internal consistency (Cronbach’s alpha, 0.94)^25^. IBSQoL questionnaire has 8 subscales including "dysphoria", "interference with activity", "body image", "health worry", "food avoidance", "social reaction", "sexual worries" and "relationship". A five-point response scale from 1 (always) to 5 (never) was used to measure sufferers’ QoL. Scores of all items and items for each subscale were summed to calculate total scores and were converted to scales between 0 to 100. Higher scores indicated better QoL.

World Health Organization QoL questionnaire (WHOQOL)-BREF, 26 items, was used to comparison QoL between 2 groups. It has acceptable validity and reliability in its Persian version^26^. Questions 1 and 2 are used to measure the overall QoL and general health, and other 24 items evaluate 4 domains such as social relationships, psychological, environmental, and physical health. The items are given a 5-point Likert scale, and each domain is determined by summing its specific items. Also, a higher score showed higher QoL.

### Statistical analysis

We used SPSS software version 24 (SPSS Inc., Chicago, IL, USA) to perform statistical analyses. In order to show the normal distribution of variables, Kolmogrov-Smirnov test was applied. Independent t test was performed to compare quantitative variables means and Chi-square test was used to compare qualitative variables between the groups. Linear correlations between continuous quantitative variables were applied using Pearson's test. To estimate the association between serum zinc levels and odds of IBS-D, logistic regression was conducted. In the adjusted model, employment, educational level, dietary zinc intake, depression, anxiety, stress, overall QoL and general health, physical health, and psychological health were controlled. Statistical significance was defined at p-value < 0.05.

### Ethical approval and consent to participate

The present study was extracted from the MSc dissertation of MR that has been approved by the Vice-Chancellor for research, Isfahan University of Medical Sciences (reg. code IR.MUI.RESEARCH.REC.1398.826).

## Results

General characteristics of participants in the two groups are described in Table 1. The employment and educational levels were significantly different between the cases and controls (p = 0.031 and 0.043, respectively). The significant differences were not observed between IBS-D patients and healthy controls in terms of age, gender, anthropometric measurements, dietary intakes of zinc, as well as other nutrients intakes. The serum zinc levels were lower in IBS-D patients than those in healthy controls (p = 0.001). Although the number of subjects with zinc deficiency was higher in IBS-D patients, it was not different between the two groups.

**Table 1:** General characteristics of the study groups.

Characteristics	IBS-D patients (n = 61)	Healthy controls (n = 61)	p-value
Age (year)	37.01 ± 8.46	37.32 ± 9.02	0.845
**Gender**			0.99
Female	38 (62.3%)	38 (62.3%)	
Male	23 (37.7%)	23 (37.7%)	
BMI (kg/m^2^)	25.08 ± 3.44	25.48 ± 2.71	0.476
Waist circumference (cm)	90.56 ± 9.63	91.31 ± 9.29	0.662
Hip circumference (cm)	101.02 ± 6.77	101.76 ± 5.87	0.52
**Employment**			0.031*
Unemployed	20 (32.8%)	14 (23%)	
Self-employment	14 (23%)	6 (9.8%)	
Physician	3 (4.9%)	0 (0%)	
Employee	17 (27.9%)	32 (52.5%)	
Worker	1 (1.6%)	2 (3.3%)	
Student	6 (9.8%)	7 (11.5%)	
**Educational level**			0.043*
Lower than diploma	16 (26.2%)	8 (13.1%)	
Diploma	9 (14.8%)	19 (31.1%)	
University	36 (59.0%)	34 (55.7%)	
Serum zinc (µg/dL)	76.12 ± 17.10	100.54 ± 49.68	0.001*
Subjects with zinc deficiency (< 70 µg/dL)	26 (42.6%)	18 (29.5%)	0.131
Dietary zinc intake (mg/day)	8.54 ± 3.09	8.54 ± 2.93	0.998
Energy intake (Kcal)	2139.23 ± 710.68	2103.76 ± 642.97	0.777
CHO^1^ intake (g)	324.77 ± 112.49	318.53 ± 98.12	0.749
Protein intake (g)	76.34 ± 26.73	75.49 ± 23.90	0.855
Fat intake (g)	62.93 ± 27.58	62.16 ± 26.22	0.877
Fiber intake (g)	16.11 ± 8.20	17.52 ± 10.49	0.418
Calcium intake (mg)	718.84 ± 302.66	755.69 ± 328.97	0.528
Iron intake (mg)	23.28 ± 10.06	22.73 ± 10.29	0.772
Copper intake (mg)	1.30 ± 0.62	1.25 ± 0.51	0.641
Magnesium intake (mg)	245.98 ± 108.94	236.27 ± 81.50	0.584

^1^Carbohydrate.

Data are presented as mean (standard deviation) or frequency (%).

Independent sample t-test was used for comparison of quantitative variables.

Chi-square test was used for comparison of qualitative variables.

*p-value < 0.05.

Table 2 demonstrates the higher scores of depression (p = 0.014), anxiety (p = 0.005), as well as stress (p = 0.001), and lower overall QoL and general health, physical health, and psychological health scores (p ≤ 0.001) among IBS-D patients compared to the healthy ones. According to the IBS-QoL questionnaire, “food avoidance” had the lowest (51.09 ± 26.80), and “relationship” had the highest score (78.14 ± 23.30) in IBS-D patients.

**Table 2:** Mood states and quality of life score of the participants.

Variables		IBS-D patients (n = 61)	Healthy controls (n = 61)	p-value
Mood states	Depression	12.32 ± 10.72	8.03 ± 8.11	0.014*
	Anxiety	10.98 ± 8.63	6.78 ± 7.55	0.005*
	Stress	19.44 ± 10.46	13.50 ± 9.44	0.001*
WHO Quality of Life	Overall QoL and general health	53.48 ± 18.97	72.74 ± 12.81	< 0.001*
	Physical health	59.59 ± 18.67	71.30 ± 13.95	< 0.001*
	Psychological health	55.80 ± 16.08	65.22 ± 12.35	< 0.001*
	Social relationships	61.19 ± 17.73	65.84 ± 14.24	0.113
	Environmental health	57.52 ± 14.01	59.52 ± 12.62	0.41
IBS Quality of Life	Total IBS-QoL	59.18 ± 24.60	–	–
	Dysphoria	60.42 ± 26.79	–	–
	Interference with activity	59.49 ± 26.88	–	–
	Body image	68.47 ± 25.80	–	–
	Health worry	53.55 ± 24.60	–	–
	Food avoidance	51.09 ± 26.80	–	–
	Social reaction	61.20 ± 24.57	–	–
	Sexual worries	66.48 ± 24.71	–	–
	Relationship	78.14 ± 23.30	–	–

Data are presented as mean (standard deviation).

Independent sample t-test was used for comparison of quantitative variables.

*p-value < 0.05.

Dietary zinc intakes and components of mood states in the two groups showed no correlation (Table 3). Dietary zinc intakes were directly correlated with psychological health scores in the controls (r = 0.295, p = 0.022) and with body image in the IBS-D group (r = 0.266, p = 0.044). Also, there was a marginal positive correlation between dietary zinc intakes and physical health score in healthy controls (r = 0.245, p = 0.06).

**Table 3:** Correlation between dietary zinc intakes with depression, anxiety, stress and quality of life.

Variables		IBS-D group (n = 58)		Control group (n = 60)	
		r	p	r	p
Mood states	Depression	0.055	0.684	−0.169	0.196
	Anxiety	−0.031	0.816	−0.138	0.291
	Stress	0.021	0.873	−0.163	0.213
WHO Quality of Life	Overall QoL and general health	0.089	0.504	0.204	0.118
	Physical health	0.167	0.209	0.245	0.060
	Psychological health	0.039	0.771	0.295	0.022*
	Social relationships	−0.152	0.256	0.157	0.232
	Environmental health	−0.003	0.983	0.144	0.273
IBS Quality of Life	Total IBS-QoL	0.090	0.501	–	–
	Dysphoria	0.136	0.310	–	–
	Interference with activity	0.006	0.962	–	–
	Body image	0.266	0.044*	–	–
	Health worry	0.049	0.714	–	–
	Food avoidance	0.077	0.565	–	–
	Social reaction	−0.040	0.763	–	–
	Sexual worries	−0.045	0.736	–	–
	Relationship	0.019	0.890	–	–

Data are presented as the Pearson’s correlation coefficient (r).

*p-value < 0.05.

Correlation between serum zinc levels with mood states and QoL is presented in Table 4. Although a significant correlation was not found, a marginally positive correlation between levels of serum zinc and social reaction, as one of the components of IBS-QoL, was observed in the IBS-D group (r = 0.241, p = 0.061).

**Table 4:** Correlation between serum zinc levels with mood states and quality of life (QOL) scores.

Variables		IBS-D group (n = 61)		Control group (n = 61)	
		r	p	r	p
Mood states	Depression	−0.085	0.514	−0.060	0.645
	Anxiety	0.072	0.579	−0.048	0.715
	Stress	−0.163	0.211	−0.038	0.772
WHO Quality of Life	Overall QoL and general health	−0.003	0.982	−0.205	0.112
	Physical health	−0.135	0.301	−0.015	0.908
	Psychological health	−0.061	0.639	−0.023	0.858
	Social relationships	−0.056	0.668	−0.034	0.793
	Environmental health	−0.033	0.802	−0.175	0.178
IBS Quality of Life	Total IBS-QoL	−0.027	0.839	–	–
	Dysphoria	−0.051	0.699	–	–
	Interference with activity	−0.032	0.809	–	–
	Body image	−0.025	0.847	–	–
	Health worry	−0.122	0.351	–	–
	Food avoidance	−0.002	0.989	–	–
	Social reaction	0.241	0.061	–	–
	Sexual worries	−0.173	0.183	–	–
	Relationship	0.024	0.853	–	–

Data are presented as the Pearson’s correlation coefficient (r).

The Logistic regression showed no significant association between zinc deficiency and odds of IBS-D in both crude and adjusted models (OR: 1.775; 95% CI: 0.840–3.751; OR: 2.41; 95% CI: 0.849–6.840, respectively) (Table 5).

**Table 5:** Logistic regression for assessing the relationship between zinc deficiency and odds of IBS-D.

	Serum zinc levels (µg/dL)			
	≥ 70		< 70	
	OR (95% CI)	p	OR (95% CI)	p
Crude	1	–	1.775 (0.840–3.751)	0.133
Model I^1^	1	–	2.41 (0.849–6.840)	0.098

^1^Adjusted for employment, educational level, zinc intake, depression, anxiety, stress, overall QoL and general health, physical health and psychological health.

## Discussion

In the previous study, we evaluated the correlation between zinc nutritional status with serum zonulin, as a serum marker of intestinal permeability, and gastrointestinal symptoms in IBS-D patients^27^. The findings of the present study revealed that the serum concentration of zinc was lower in patients compared to their controls. The intestine is the main route of absorption and excretion of zinc. Decreased absorption, as well as increased fecal excretion of zinc, and gut dysbiosis might be considered as potential causes of low levels of serum zinc in IBS patients. Gut dysbiosis can alter the functional capacity of the microflora, resulting in decreased mineral absorption, carbohydrate digestion and fermentation, and short-chain fatty acid production. These effects may reduce zinc bioavailability and disturb GI and mental health^28–30^. Current investigation showed that the mood states and QoL were impaired in IBS-D patients. Although, a significant positive correlation was observed between dietary zinc intakes and psychological health in the control group, the other components of the QoL were not correlated with dietary intakes of zinc in both groups. Moreover, IBS-QoL, showed that body image was positively correlated with dietary intakes of zinc. The aforementioned reasons may be involved in this correlation. However, there was no significant correlation between serum zinc and components of mood states and QoL.

In agreement with our findings, a preliminary study by Cavallo et al. reported low serum zinc levels and increased fecal excretion of zinc in two subgroups of IBS patients^15^. In contrast, other studies on patients with IBS showed no significant differences in serum levels of zinc between the cases and controls^31,32^. Although our finding indicated no differences in dietary zinc intakes between the groups, some studies reported that dietary intake of zinc in IBS patients was lower than those of healthy subjects^16,17^.

Irritable bowel syndrome is not a life-threatening disease, however, it can affect the normal life of patients. Our findings illustrated that the lower QoL and higher severity of depression, anxiety, and stress are related to IBS-D. Some studies suggested that the QoL score was significantly lower in IBS patients compared to their healthy subjects^33,34^. Physical discomfort, food avoidance, sexual problems, psychological disease, sleep disorders, fatigue, and functional disorders can contribute to the declined QoL of these patients^35^. A meta-analysis by Zhang et al. showed that symptoms of depression were greater in IBS patients in comparison to the healthy ones^36^. In agreement with our findings, two studies that assessed mood status by DASS questionnaire observed that the scores of stress, anxiety and depression were higher in IBS patients^37,38^. Alteration in intestinal microbiota and abnormal function of the gut-brain axis are possible mechanisms for mood alterations in IBS^39,40^.

According to our results, there was a direct correlation between dietary zinc intake and psychological health in the control group and body image in the IBS-D group. In contrast to our findings, previous studies found a significant association between zinc nutritional status with mood and QoL. A case–control study in Iran conducted on the major depressive disorder (MDD) female students reported a linear inverse correlation between dietary and serum zinc levels and Beck’s scores^41^. In addition, review by Elise et al. demonstrated that zinc deficiency was associated with mood disorders and supplementation with zinc might have beneficial role in the treatment of these disorders^42^. Regarding the QoL and zinc status, a double-blind randomized and placebo-controlled trial reported that supplementation with zinc sulfate had a beneficial effect on improving the health-related QoL and its components in women with premenstrual syndrome^43^. However, a cross-sectional study conducted on 297 elderly subjects indicated no significant association between dietary or serum zinc and anxiety. Noteworthy, low serum zinc levels, but not dietary zinc, were associated with higher odds of depression^44^. Differences in dietary assessment tools, sample size, and health status of the participants might be regarded as the sources of inconsistency in studies results.

As the possible underlying mechanisms, higher receptors of serotonin in the hippocampus and frontal cortex, modulation of glutamate signaling, elevated concentration of brain-derived neurotrophic factor (BDNF) in the hippocampus and cortex, and antioxidant activity of zinc are proposed for the antipsychotic effects of this trace element^45,46^. Therefore, impaired zinc homeostasis can lead to states of cognitive, emotional, and behavioral problems.

The result of our study indicated that the association between zinc deficiency and odds of IBS-D might be confounded by variables such as employment and educational status, dietary zinc intake, components of QoL, and general health status. Therefore, with adjustment for these confounding variables, the association of zinc deficiency with IBS-D odds became stronger compared to the crude model.

This study had some strengths. To the best of our knowledge, this is the first study that has investigated the correlation of zinc nutritional status with mood disorders and QoL in IBS-D patients. In addition, to prevent alteration in common dietary intakes and food habits, newly diagnosed IBS-D patients were considered as the cases group.

As a limitation, we did not use a semi-quantitative FFQ to assess the usual dietary intakes during the last year. Also, we were unable to measure other biomarkers such as serum metallothionein concentrations.

## Conclusion

In the present study, serum zinc levels, overall QoL, physical and psychological health were lower and scores of depression, anxiety, and stress were higher in IBS-D patients. In addition, higher dietary intake of zinc correlated with improvements in psychological health and body image in the controls and cases, respectively. Further research with prospective and clinical trial design is warranted to illustrate any causal relationship.

## Data Availability

The datasets generated and/or analysed during the current study are not publicly available as per the rules and regulations of the Department of Clinical Nutrition at the Isfahan University of Medical Science but are available upon reasonable request from the corresponding author.
